# From data to action: the FRAME conceptual framework for translating consumer wearable feedback into behavior change support

**DOI:** 10.3389/fdgth.2026.1871670

**Published:** 2026-09-15

**Authors:** Sundus Malaikah

**Affiliations:** 1 Department of Clinical Nutrition, Faculty of Applied Medical Sciences, King Abdulaziz University, Jeddah, Saudi Arabia; 2 Performance, Exercise, and Health Sciences Unit, King Fahad Medical Research Center, King Abdulaziz University, Jeddah, Saudi Arabia

**Keywords:** behavior change, conceptual framework, digital health, health promotion, narrative review, physical activity, self-monitoring, wearable devices

## Abstract

**Background:**

Consumer wearables are now widely used across health promotion, wellness, and recreational fitness. Although wearable-supported interventions can improve physical activity and related outcomes, effects are typically modest, heterogeneous, and more consistent when devices are embedded within broader behavior change support rather than used alone. In applied settings, however, wearable use often remains focused on metric display and passive monitoring, leaving practitioners without a clear framework for translating data into behaviorally meaningful support.

**Objective:**

This paper proposes FRAME, a theory-informed conceptual framework intended to help practitioners interpret consumer wearable outputs as behaviorally relevant signals rather than as self-sufficient indicators of progress or failure.

**Methods:**

The framework was developed through a targeted narrative synthesis of literature on wearable-supported interventions, self-monitoring, feedback design, engagement, adaptive support, and applied behavior change theory. The purpose of the synthesis was not to estimate pooled effects, but to identify recurring interpretive and implementation problems that arise when wearable data are used in real-world behavior support encounters.

**Framework overview:**

FRAME comprises five linked steps: Filter the signal, Read the behavior pattern, Align a behavior change target, Message and micro-plan, and Evaluate and iterate. Across these steps, wearable data are treated as inputs into interpretation, hypothesis generation, collaborative planning, and iterative review. The framework is grounded primarily in self-regulation, the Capability-Opportunity-Motivation-Behavior (COM-B) model, self-determination theory, social cognitive theory, habit formation, and relapse prevention.

**Conclusion:**

For practitioners, the framework offers a structured way to judge whether a wearable signal is usable, what behavior pattern it may reflect, which target to prioritize, and how to discuss the data in an autonomy-supportive way. For wearable users, this is intended to yield more collaborative and sustainable behavior change support.

## 1 Introduction

Consumer wearables, including wrist-worn accelerometers, smartwatches, fitness bands, and biosensor-based devices such as continuous glucose monitors (CGMs), have become increasingly visible in health promotion, wellness, and recreational fitness (1, 2). Step counts, active minutes, sleep summaries, heart-rate trends, and device-generated recovery or readiness metrics are now routinely presented to users and, increasingly, to practitioners working in behavior support contexts (3, 4). As access to these data has expanded, the central challenge has shifted from data generation to data interpretation: not whether wearable outputs can be produced, but how they might be translated into meaningful support for behavior change (4, 5).

The current evidence base supports a cautious but potentially useful role for wearables in behavior change. Systematic review and meta-analytic evidence indicate that consumer-based wearable activity trackers may improve physical activity outcomes, particularly daily step count and moderate-to-vigorous physical activity, although average effects are generally modest and vary across populations and intervention designs (6). More recent review-level evidence similarly suggests that wearable activity trackers can support improvements in physical activity and related outcomes, but that effects are more consistent when wearables are embedded within multicomponent interventions that include counseling, goal setting, feedback, or other behavior change techniques (1). At the same time, user engagement with wearable platforms often declines over time, even when data remain available, underscoring that access to data alone is unlikely to sustain device-supported improvements in physical activity and related behaviors (1, 7).

Taken together, these findings suggest that consumer wearables may be better understood as tools that support self-monitoring, prompting, and review than as interventions in themselves. Yet this observation leaves a practical question unresolved: what should a practitioner actually do with wearable data once those data are available? Between observing a signal and selecting a next step lies a translation task that is often under-specified in the literature. A low-step week, irregular sleep pattern, incomplete tracking record, or declining recovery score may all appear actionable, but the behavioral meaning of such outputs remains uncertain without contextual interpretation.

Existing research has advanced several adjacent areas relevant to this problem. Work on digital behavior change interventions has clarified the importance of engagement, self-monitoring, and feedback processes (7). Just-in-time adaptive intervention (JITAI) research has helped specify how adaptive support may respond to changing context over time (8). Implementation-oriented work has also begun to address the challenge of integrating patient-generated or wearable-derived data into practice workflows (4, 5, 9). However, these literatures do not by themselves provide a practitioner-facing decision heuristic for moving from wearable output to behaviorally coherent support during brief real-world encounters.

The present paper addresses that gap by proposing FRAME, a conceptual framework for translating consumer wearable outputs into behavior change support. FRAME is not intended as a checklist for device procurement, a theory of digital engagement, a JITAI architecture, or a clinical decision protocol. Rather, it is proposed as a structured interpretive workflow that helps practitioners decide whether a wearable signal is usable, what kind of behavior pattern it may reflect, which target appears most feasible, and how the resulting conversation might be framed.

FRAME consists of five linked steps: Filter the signal, Read the behavior pattern, Align a behavior change target, Message and micro-plan, and Evaluate and iterate. The framework draws on six behavior change perspectives selected for their practical relevance to interpretation and support decisions: self-regulation, the Capability–Opportunity–Motivation–Behavior (COM-B) model, self-determination theory (SDT), social cognitive theory (SCT), habit formation, and relapse prevention (10–14). These theories are not presented as competing explanations, but as complementary lenses that illuminate different points in the translation process.

By articulating this workflow, the paper aims to shift wearable-supported practice away from metric-centered monitoring and toward behavior-centered interpretation. The contribution is therefore conceptual and translational: FRAME offers a structured way of thinking about how wearable data may inform support, while remaining explicit about the evidentiary and practical limits of consumer-generated metrics.

## 2 Methods

### 2.1 Study design

This paper presents a conceptual framework developed through a targeted narrative synthesis (15). The design was interpretive in nature and its objective was not to produce a comprehensive systematic review or pooled estimate of intervention effects, but to identify and explain recurring interpretive and implementation problems in wearable-supported behavior change practice and to map these problems onto behavior change theory in order to construct a coherent practitioner-facing model. Narrative and critical review designs are appropriate when the goal is conceptual development and explanation rather than exhaustive evidence aggregation (15, 16). The framework was developed for consumer wearables used in health promotion, wellness, recreational fitness, and clinically adjacent lifestyle-support contexts in which wearable outputs may inform behavior-focused conversations but do not independently determine diagnosis or treatment.

### 2.2 Review question

The synthesis was guided by the following question: How does the existing literature inform a structured, behavior-science-informed workflow for translating consumer wearable outputs into practitioner-facing behavior support decisions, and what recurring interpretive problems must such a workflow address?

### 2.3 Search approach

Literature identification was conducted iteratively using three complementary strategies. First, targeted searches were undertaken in PubMed, PsycINFO, and Scopus using combinations of terms related to consumer wearables, activity trackers, self-monitoring, feedback, engagement, attrition, adaptive interventions, implementation, and behavior change. Searches were oriented toward review-level evidence and key conceptual papers published across digital health, behavioral medicine, and health behavior change research. Searches were conducted iteratively during framework development, with the final search completed in January-February 2026; no lower publication-date limit was applied because foundational theoretical work predates the consumer wearable literature. Second, citation chaining was applied to influential reviews and foundational articles to identify additional sources. For this purpose, reviews were considered influential when they provided review-level or umbrella-level syntheses directly addressing wearable-supported behavior change, engagement, attrition, or feedback design; articles were considered foundational when they represented primary statements, validations, or authoritative reviews of the behavior change theories and models applied in the framework. Third, theory papers were purposively included when they offered direct explanatory value for the interpretive decisions practitioners face when working with wearable data.

Because the goal of the paper was framework development rather than evidence aggregation, formal protocol registration, duplicate screening, and risk-of-bias appraisal were not undertaken. Instead, sources were selected for conceptual relevance, practice relevance, and explanatory contribution to the framework architecture. This approach is consistent with prior framework-oriented synthesis work in digital behavior change research (7).

### 2.4 Eligibility criteria and source selection

Sources were eligible for inclusion when they met all of the following criteria: (1) they addressed consumer wearables or closely related digital self-monitoring technologies used in health-promotion, wellness, fitness, or lifestyle-support contexts; (2) they provided review-level evidence, conceptual analysis, theory, or implementation guidance relevant to at least one of the five evidence domains described below; and (3) they offered direct explanatory value for the interpretive or support decisions practitioners face when working with wearable data. Sources were excluded when they focused primarily on device validation or brand-level technical comparison, signal-processing or algorithm development, elite sport performance analytics, or high-acuity clinical monitoring without clear relevance to general behavior-support practice. Peer-reviewed publications were prioritized, with emphasis on review-level evidence where available.

All searching, screening, source selection, and data extraction were conducted by the sole author. Inclusion decisions were revisited iteratively throughout framework development, and sources were retained only when they continued to contribute explanatory value to at least one framework component. In total, 29 sources informed the synthesis. Table 1 summarizes the 14 key evidence sources that most directly informed FRAME across five evidence domains; additional theoretical, conceptual, and implementation-oriented sources are cited in the text. This approach is consistent with prior framework-oriented synthesis work in digital behavior change research (7).

**Table 1:** Summary of evidence informing the FRAME approach.

Reference (Study Type)	Population, setting & device type	Behavior change support	Key finding
Domain 1: Wearable-Supported Intervention Effectiveness			
Brickwood et al., 2019 (6) Systematic review & meta-analysis 23 trials	Adults; health promotion and clinical settings *Fitbit, Jawbone, Polar, BodyMedia, Withings Pulse (mixed)*	Self-monitoring; app-based feedback; multifaceted subgroup added counseling and goal-setting	Multifaceted interventions produced effects ∼50% larger than device alone (steps: SMD 0.24; MVPA: SMD 0.27); sedentary behavior non-significant; structured support is necessary, not supplementary.
Li et al., 2021 (28) Systematic review & meta-analysis 44 trials	Adults; general and clinical populations *Fitbit, Mi Band, Apple Watch, Garmin (mixed)*	Self-monitoring; expert support (counselor, coach, or clinician) in subgroups; variable personalization	Wearables improved structured exercise but not habitual activity; expert support type and intervention duration independently moderated effects.
Ferguson et al., 2022 (1) Umbrella review 39 SRs; *n* = 163,992	Adults; clinical and non-clinical populations *Fitbit, Garmin, Apple Watch, Polar, Jawbone (mixed across SRs)*	Variable: feedback, counseling, goal-setting, app-based support across included SRs	Effects modest and heterogeneous (PA SMD 0.3–0.6); engagement declines regardless of device brand; wearables most effective as components of broader behavior support.
Longhini et al., 2024 (2) Umbrella review 51 SRs; 302 unique trials	Adults ≥18; general and clinical populations *Fitbit most common; mixed consumer and research-grade devices*	Variable and poorly reported; self-monitoring and goal-setting most common	Steps and MVPA improved in middle-aged adults (moderate certainty); sedentary behavior inconsistent; role of additional intervention components underreported; maintenance evidence weak.
Lai et al., 2023 (29) Systematic review & meta-analysis of RCTs; *n* = 1,608	Adults ≥18 years; mixed clinical and community settings across 9 countries; smartwatches, sleep position trainers, wearable respiratory monitoring devices, virtual reality headsets (mixed)	Goal setting; real-time feedback; reminders; health coaching; social support; self-monitoring of sleep parameters	Wearable-delivered interventions significantly reduced sleep disturbance (ES = 0.37) and sleep-related impairment (ES = 1.06); improved oxygen desaturation and respiratory distress indices; effects attributed to self-monitoring, feedback, and coaching components rather than device feedback alone.
Domain 2: Behavior Change Technique Coverage in Consumer Trackers			
Lyons et al., 2014 (3) Systematic content analysis 13 electronic activity monitors	Adults; consumer and health promotion contexts *Fitbit, Jawbone UP, Nike* *+* *FuelBand, Basis, Misfit, Bodymedia, BodyBugg, DirectLife, Gruve, Striiv, Omron, Moves app, Withings*	Device-embedded BCTs coded using the 93-item BCT taxonomy; no practitioner support component	Devices contained a mean of 23 BCTs, concentrated in self-monitoring and feedback; action planning, graded tasks, problem solving, and relapse prevention were consistently absent across devices.
Düking et al., 2020 (17) Content analysis 5 wrist-worn consumer wearables	Adults; wellness and fitness contexts *Fitbit Versa, Garmin Vívoactive 3, Apple Watch Series 3, Polar M600, Xiaomi Amazfit Stratos 2*	BCTs coded using BCTTv1 (93-item taxonomy); no practitioner support component	Mean of 19 BCTs per device; barrier identification/problem solving absent in all five devices; no device included self-talk, commitment, or information about emotional consequences — key techniques for sustained behavior change.
Domain 3: Feedback Design and Adaptive Support Logic			
Schembre et al., 2018 (25) Systematic review & practice framework 31 studies	Mixed; diet and physical activity intervention contexts *Body-worn sensors, Fitbit, Jawbone, wrist-worn accelerometers, dietary sensors (mixed)*	Just-in-time feedback on PA, sedentary behavior, and dietary intake; variable theoretical grounding	Feedback content, framing, and delivery timing influence outcomes independently of data access; theory-informed design matters over metric display alone.
Krukowski et al., 2024 (26) Systematic review & meta-analysis 19 studies; *n* = 3,261	Adults; behavioral weight management and diet/PA contexts *App-based and wearable-assisted tracking platforms (mixed)*	Practitioner-generated and algorithm-generated feedback on self-monitoring data; variable personalization	Feedback generation type and presentation format independently affect engagement and outcomes; practitioner-crafted feedback outperforms algorithm-generated messages.
Hardeman et al., 2019 (30) Systematic review of JITAIs	Adults; mixed health behavior contexts *Smartphones paired with wearable sensors; Fitbit, accelerometers, heart rate monitors (mixed)*	Real-time adaptive support triggered by sensor data; prompts, goal-setting, self-monitoring	Adaptive context-sensitive support outperforms fixed-schedule feedback; practitioner-facing decision rules are rarely well-specified in published JITAI protocols.
Domain 4: Engagement, Attrition, and User Experience			
Attig & Franke, 2020 (18) Review + empirical study *n* = 159 former users	Adults; general consumer populations; former tracker users *Fitbit and Jawbone most referenced; multiple brands in survey*	Not applicable — abandonment study; no active intervention components	Abandonment driven by loss of motivation, perceived data irrelevance, usability friction, and routine disruption — not simple non-compliance; disengagement is predictable and multi-causal.
Keats et al., 2023 (31) Engagement and user experience review	Adults; wellness and fitness contexts *Fitbit, Apple Watch, Garmin (most frequently referenced)*	Variable; app-based and practitioner-supported interventions	Engagement declines predictably after the novelty phase across device types; practitioner-led support can buffer decline; re-engagement planning is necessary from the outset.
Domain 5: Practitioner Implementation and Workflow Integration			
Sadhu et al., 2023 (5) Implementation and qualitative review	Practitioners and patients; real-world and hybrid care programs *Mixed consumer wearables; Fitbit most referenced; CGM where applicable*	Variable; practitioner-mediated interpretation and consultation support	Interpretation burden and usability friction are recurring barriers regardless of device type; raw wearable outputs are not workflow-ready and require structured translation guidance.
Szeto et al., 2024 (4) Implementation guidance; WATCH checklist development	Clinicians and practitioners; low-resource and hybrid care settings *Fitbit, Apple Watch, Garmin; CGM in hybrid contexts*	Practitioner-facing workflows; remote and asynchronous support models	Practitioner translation workflows remain underdeveloped; existing checklists address reporting requirements but not behavior-science decision logic.

Presents the key evidence sources that most directly informed the framework; the full synthesis additionally drew on the theoretical and conceptual literature cited in the text (see Section 2). Device types listed in italics reflect the most commonly reported devices in each study; mixed indicates variable devices across included trials.

BCT, behavior change technique; CGM, continuous glucose monitor; JITAI, just-in-time adaptive intervention; MVPA, moderate-to-vigorous physical activity; PA, physical activity; SMD, standardized mean difference; SR, systematic review; WATCH, Wearable Activity Tracker Checklist for Healthcare.

### 2.5 Analysis and framework development

Analysis followed a structured conceptual mapping process conducted in four stages. First, included sources were read in full and annotated for recurring interpretive and implementation problems reported in wearable-supported behavior change practice. Data were screened at the level of recurring interpretive challenges, support implications, and theory-relevant decision points, rather than at the level of individual effect estimates; this approach was chosen because the review question concerned how practitioners should interpret and act on wearable outputs. Second, annotated problems were grouped inductively into six recurring challenge areas: signal ambiguity, non-wear or missingness, declining engagement, overreliance on raw metrics, uncertainty regarding causal interpretation, and difficulty converting observed patterns into feasible next steps. This grouping was conceptually oriented and iterative, resembling thematic organization of conceptual content rather than formal qualitative coding of primary data. Third, each challenge area was mapped onto the behavior change theory or model judged to offer the most direct explanatory and practical response.

Theories were selected from the foundational literature on the basis of (a) established application to physical activity and lifestyle behavior, (b) explanatory fit with the challenge in question, and (c) practical usability within brief consultations. Through this process, self-regulation provided the overall logic of iterative review and adjustment; COM-B informed behavioral diagnosis; self-determination theory (SDT) informed communication style and autonomy support; social cognitive theory (SCT) contributed a lens on confidence and mastery; habit formation informed cue-linked repetition and routine stability; and relapse prevention informed lapse interpretation and re-engagement. Fourth, the resulting challenge–theory pairings were organized into five sequential but iterative steps, each constructed to address a distinct decision problem in the translation process. Table 1 summarizes the evidence domains and practice-relevant lessons that informed development of FRAME; the framework itself represents a theory-informed synthesis of these inputs rather than a structure directly tested in prior studies.

## 3 Results

### 3.1 Conceptual foundation

The following sections present the conceptual findings of the synthesis, organized around the evidence domains summarized in Table 1: why wearable data require translation into behavior support, what consumer wearable outputs can and cannot support, and the theoretical foundations on which FRAME rests.

#### 3.1.1 Why wearable data require translation into behavior support

Consumer wearables can contribute to behavior change, but typically not through data provision alone. Their most plausible contribution lies in supporting self-monitoring, prompting attention, and enabling review of behavior over time. These functions are important, but they are not equivalent to behavior change support in a fuller sense. Evidence from wearable intervention research suggests that effects are generally more consistent when devices are integrated into broader support structures that include planning, counseling, goal setting, or feedback (1, 2, 6).

From a self-regulation perspective, wearable outputs may help users compare current behavior against a desired standard and detect discrepancies that warrant adjustment (10). However, discrepancy detection does not by itself specify what the discrepancy means or how a practitioner should respond. A low-step day may reflect time scarcity, fatigue, disrupted routine, illness, competing priorities, low confidence, or disengagement from the device itself. Similar numeric outputs may therefore carry very different behavioral implications.

For this reason, the central challenge is not merely access to data, but translation from signal to explanation and from explanation to feasible action (5). This translation problem matters because engagement with digital tools is multidimensional, unstable, and context dependent. Access to data does not ensure attention, interpretation, or sustained use (Perski et al., 7). Likewise, JITAI research indicates that support is more likely to be useful when it is context-sensitive and timely rather than uniformly delivered in response to raw metrics alone (8).

The present framework therefore adopts a behavior-centered interpretation stance. Repeated patterns, routine disruptions, plan–behavior gaps, and context-linked irregularities are treated as more informative than isolated fluctuations. In this framing, wearable outputs are not taken as definitive evidence of physiology, motivation, or adherence. Rather, they are treated as prompts for inquiry, hypothesis generation, and collaborative problem-solving.

#### 3.1.2 What consumer wearable data can and cannot tell practitioners

Consumer wearables commonly provide outputs that appear immediately actionable, including step counts, active minutes, heart-rate summaries, sleep metrics, streaks, estimated recovery scores, and fitness indicators (3, 17). In health-promotion and recreational fitness settings, such data may help identify broad patterns such as routine consistency, timing of activity, probable non-wear, repeated missed sessions, or irregular sleep timing. These patterns may support hypothesis generation regarding barriers, opportunities, and self-regulatory processes.

At the same time, the inferential limits of these outputs are substantial. Consumer wearable data are not equivalent to clinical diagnostic measures, and model-derived summaries such as readiness scores, stress scores, or estimated VO_2_max should not be interpreted as direct windows into underlying mechanism or cause. Accuracy varies according to metric, device, sensor location, user characteristics, context, and algorithm design (2, 6). Missingness is similarly ambiguous since a data gap may reflect non-wear, syncing failure, competing demands, selective disengagement, or simply low relevance to the user on that day (18).

Accordingly, FRAME treats several interpretive assumptions with caution. First, repeated patterns are generally more informative than single-day fluctuations. Second, missingness is treated as ambiguous rather than automatically equated with non-compliance. Third, model-generated scores are approached as prompts for discussion rather than as authoritative judgments. Fourth, concerning or unusual patterns may justify further review or referral, but consumer-generated data alone are not treated as sufficient diagnostic evidence.

The relational dimension is equally important. The same data stream can be used either to facilitate collaborative reflection or to amplify surveillance, pressure, and self-judgment (11, 14). Interpretation quality therefore cannot be separated from communication quality. For this reason, the framework embeds message framing within the workflow itself rather than treating communication as an afterthought.

#### 3.1.3 Theoretical foundations of FRAME

FRAME draws on six behavior change perspectives. Each contributes specific constructs to the framework rather than serving as a global explanatory account, and each is invoked at the point in the workflow where its constructs offer the most direct interpretive or practical value.

Self-regulation approaches conceptualize behavior change as a feedback loop in which individuals set standards, monitor their behavior, compare behavior against those standards, and adjust in response to detected discrepancies (10). FRAME adopts this loop as its overall architecture: wearable outputs are treated as monitoring inputs, and the five steps operationalize the compare–interpret–adjust cycle across repeated contacts, culminating in the review logic of Step 5.

COM-B model proposes that behavior occurs when capability, opportunity, and motivation are jointly sufficient, and that support should target whichever component is deficient (10, 13). FRAME applies COM-B as its primary diagnostic lens in Step 2, guiding whether an observed pattern is more plausibly read as a capability, opportunity, or motivation problem before any target is selected.

Self-determination theory (SDT) distinguishes autonomous from controlled motivation and holds that support for autonomy, competence, and relatedness fosters more sustainable behavioral engagement (11, 14, 19). FRAME applies SDT chiefly to the communication component of Step 4 and to the corresponding design principle, so that data are discussed in ways that preserve choice and invite collaboration rather than imposing judgment.

Social cognitive theory (SCT) emphasizes reciprocal interaction among personal, behavioral, and environmental factors, with self-efficacy as a central determinant of initiation and persistence (12, 20). FRAME draws on SCT in Steps 2 to 4, where fragile confidence is treated as a plausible reading of lapse-linked patterns and where plans are framed to build mastery and perceived feasibility rather than performance pressure.

Habit formation theory explains the maintenance of behavior through cue–response associations that strengthen with context-consistent repetition (21, 22). In FRAME, habit constructs inform the interpretation of routine instability in Step 2 and the preference in Step 3 for targets that stabilize timing, context, and repeatability over targets defined purely by metric magnitude.

Relapse prevention conceptualizes lapses as expected, information-rich events shaped by high-risk situations and by reactions to perceived failure, rather than as evidence that change has failed (23, 24). FRAME applies this perspective in Steps 2 and 5, where all-or-nothing patterns and post-lapse disengagement are interpreted constructively and used to plan re-engagement.

These perspectives were combined by function rather than fused into a single explanatory model: each theory is invoked at the decision point in the workflow where its constructs offer the most direct interpretive or practical value. Self-regulation supplies the overall iterative architecture; COM-B supplies the diagnostic vocabulary of Step 2; SDT and SCT jointly shape the communication and planning logic of Step 4; habit formation and relapse prevention inform how routine instability and lapses are read in Steps 2, 3, and 5. Figure 1 summarizes this alignment between the recurring interpretive challenges identified in the synthesis, the theoretical lenses applied, and the framework steps in which each lens operates.

**Figure 1:**
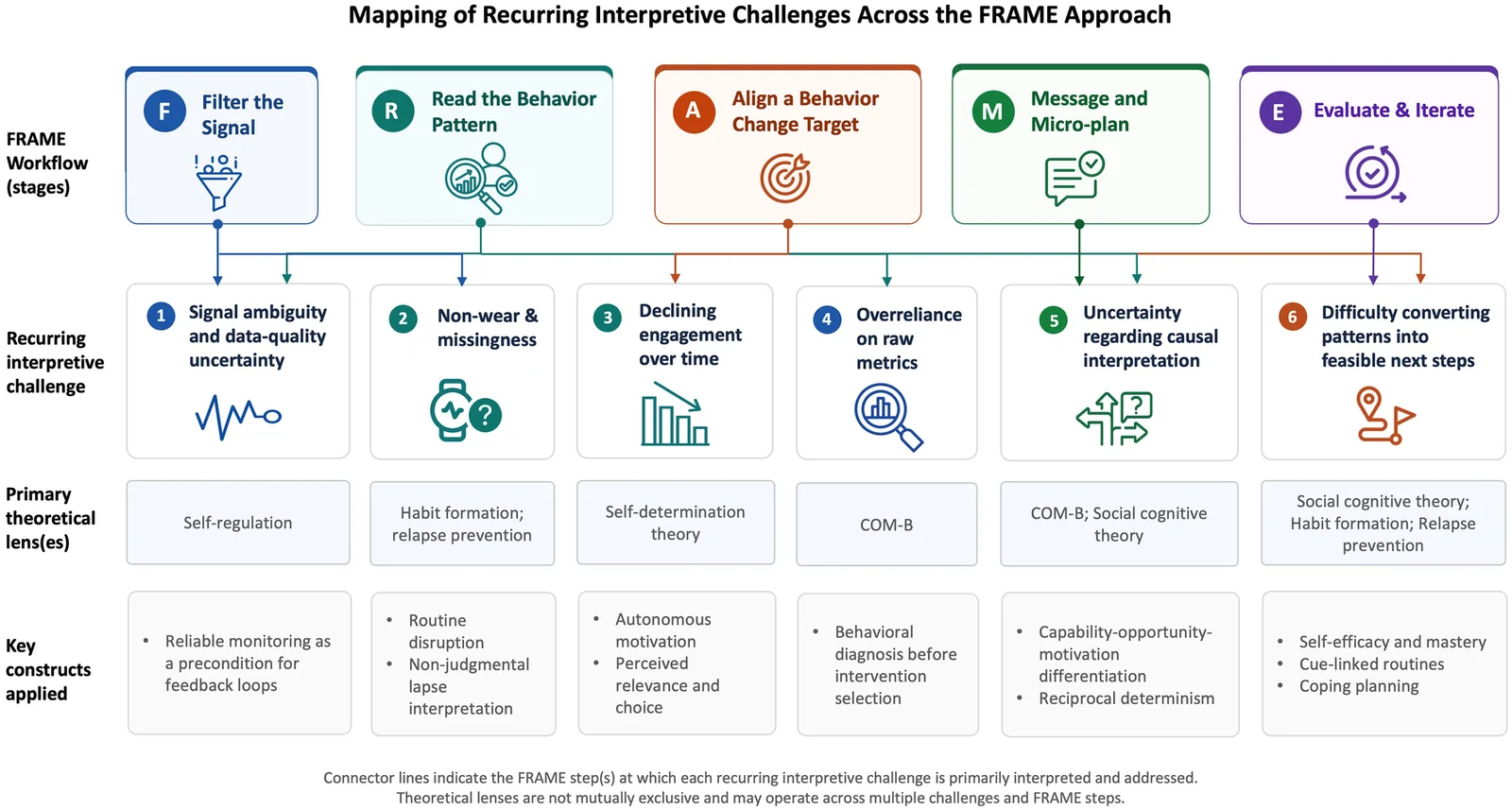
Mapping of recurring interpretive challenges to theoretical lenses and FRAME steps. Challenges were identified inductively from the reviewed literature (see Section 2); lenses reflect the primary theory applied, although steps may draw on multiple perspectives in practice. COM-B, Capability–Opportunity–Motivation–Behavior model. Icons from Flaticon: “Selective”, “Descision Making”, “Destination” and “Iteration” by Magnific, “Inspection” by zero_wing, “Target” by Uniconlabs, “Message” by adrianadam, “Signal” by Swifticons, “Smartwatch” by Ch.designer, “Graph” by Smashicons, “Searching” by IconMarketPK, licensed under Flaticon License.

### 3.2 Scoping considerations before applying FRAME

Before applying the five-step workflow, three scoping questions help determine whether wearable data are appropriate to use at all in a given interaction. These questions are not an additional step in FRAME, but a boundary-setting orientation for use.

#### 3.2.1 Are the behavioral goals clear and appropriately scoped?

The practitioner should clarify the aim of wearable use in the person's own terms. For example, supporting a walking habit, improving sleep regularity, increasing day-to-day movement, or monitoring consistency of an existing routine. Clarifying the aim helps separate health-promotion or lifestyle-support uses from disease-management questions that exceed what consumer wearable outputs can answer.

#### 3.2.2 Is wearable use psychologically appropriate for this individual at this time?

Some patterns of wearable use may be counterproductive rather than supportive. Compulsive checking, distress when goals are missed, perfectionistic responses to data, or signs of disordered exercise or eating may indicate that metric-focused engagement is doing harm. In such cases, de-emphasizing device metrics, simplifying feedback exposure, or shifting to non-metric targets may be more appropriate than intensifying tracking.

#### 3.2.3 Is the metric sufficiently fit for the decision being considered?

Consumer wearables vary in accuracy across metrics and settings (2, 6). The relevant question is not whether a device is valid in the abstract, but whether the specific output is sufficiently trustworthy for the behavioral inference under consideration. When metric quality is uncertain, the device may still be useful for trend awareness or engagement, but less suitable for making precise recommendations.

When these scoping considerations are satisfactory, FRAME may be used as an interpretive workflow. When they are not, the most appropriate action may be to reframe the device's role, simplify its use, or temporarily pause data-driven goal setting.

### 3.3 The FRAME approach

#### 3.3.1 Design principles

FRAME is guided by six design principles derived from the synthesis. Each principle is the author's formulation, but each is anchored in the literature reviewed above, and together they shape the logic of the workflow and distinguish it from device-centric models of wearable use.

##### 3.3.1.1 Pattern before prescription

Raw step counts, sleep scores, and heart-rate data are more informative when interpreted as patterns over time rather than as isolated values. A single low-output day may have limited explanatory value, whereas recurrent weekday–weekend differences, repeated missed sessions, or persistent irregularity may better indicate an actionable process (25, 26).

##### 3.3.1.2 Context before conclusion

Wearable outputs rarely include the situational, emotional, or social context needed for confident interpretation. Contextual inquiry therefore precedes strong inference. Stress, caregiving load, work structure, travel, illness, or shifts in routine may all shape the pattern that the device records but does not explain (8, 18).

##### 3.3.1.3 Behavior target before metric target

Consumer wearables emphasize numbers, but behavior support requires focus on the process likely to produce change. In many cases, the more useful question is not how to increase a score directly, but which underlying behavior such as timing, consistency, routine protection, or self-monitoring adherence, appears most feasible to target next (3, 27).

##### 3.3.1.4 Autonomy-supportive communication

Interpretation is never neutral in practice. How wearable data are discussed can shape motivation, defensiveness, and engagement. FRAME therefore encourages language that invites reflection, collaboration, and choice over language that implies surveillance or control (11, 14).

##### 3.3.1.5 Small next steps with short review cycles

Behavioral instability is often easier to address through small, reviewable adjustments than through large aspirational targets. Short review windows make it easier to observe whether a proposed change was feasible, meaningful, or misaligned with context (21, 25).

##### 3.3.1.6 Iterative adjustment over perfection

FRAME assumes that first interpretations are provisional. The goal is not to infer the true cause of a pattern with certainty, but to develop a plausible working hypothesis, test a feasible response, and revise that hypothesis through subsequent review (10, 24).

Together, these principles orient wearable-supported practice toward interpretation, dialogue, and iterative support rather than metric surveillance. Figure 2 provides an overview of the FRAME workflow and its iterative logic.

**Figure 2:**
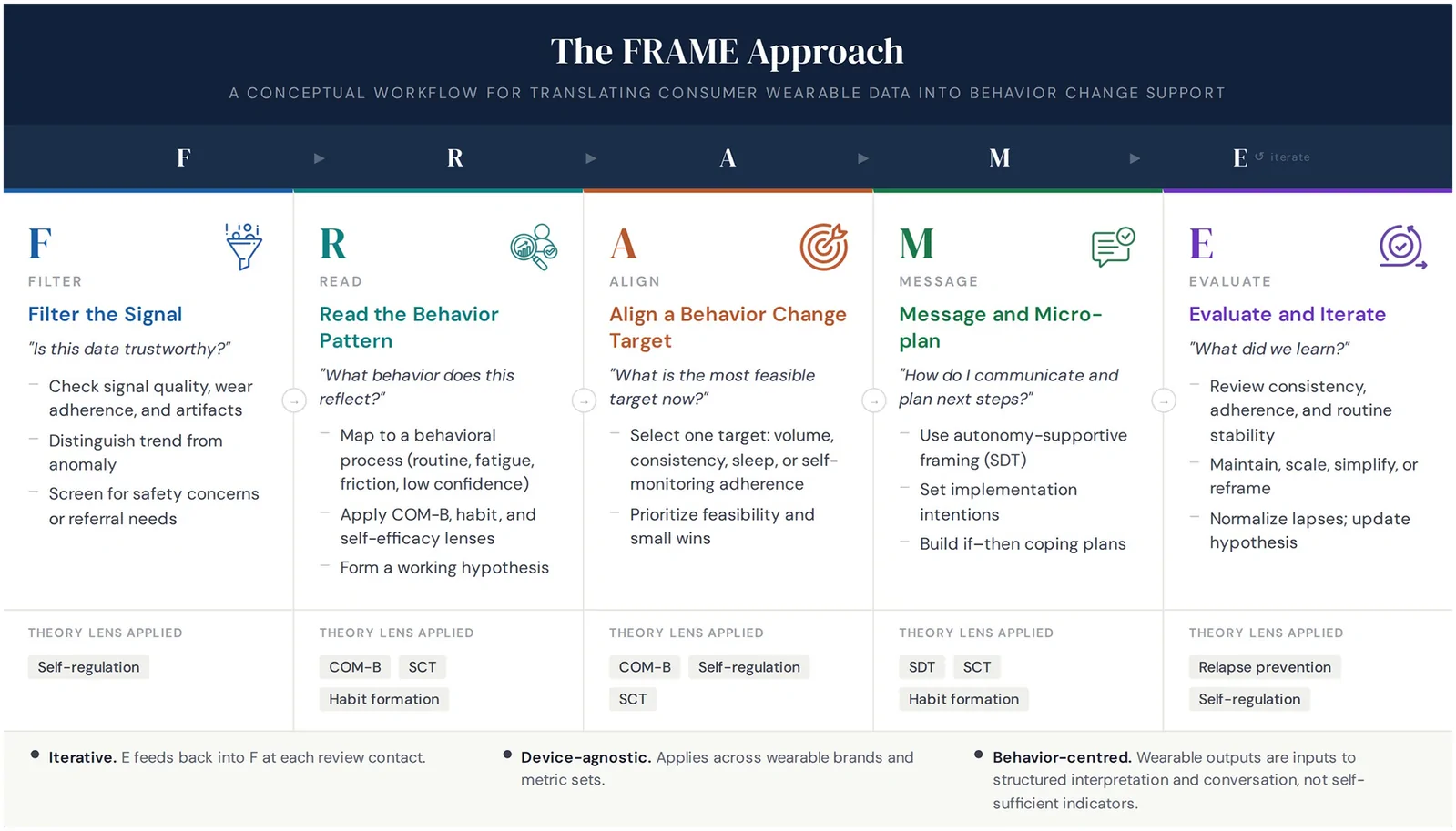
The FRAME approach: a five-step conceptual workflow for translating consumer wearable data into behavior change support. The workflow progresses from Filter the signal **(F)** to Read the behavior pattern **(R)**, Align a behavior change target **(A)**, Message and micro-plan **(M)**, and Evaluate and iterate **(E)**. The final step feeds back into the first, reflecting the framework's iterative logic across repeated contacts. Each step is informed by a relevant behavior science lens, including self-regulation, COM-B, social cognitive theory, self-determination theory, habit formation, and relapse prevention. The framework is intended for use with consumer wearable data in health-promotion, wellness, and related behavior-support contexts. COM-B, Capability, opportunity, motivation–behavior model; SCT, social cognitive theory; SDT, self-determination theory. Icons from Flaticon: “Selective” and “Iteration” by Magnific, “Inspection” by zero_wing, “Target” by Uniconlabs, “Message” by adrianadam, licensed under Flaticon License.

#### 3.3.2 FRAME steps

##### 3.3.2.1 Step 1: filter the signal

The first task is to determine whether the available data are sufficiently interpretable to support discussion. This includes checking likely wear adherence, syncing gaps, obvious anomalies, implausible values, and the general coherence of the pattern (5, 18). At this stage, the aim is not to interpret behavior deeply, but to decide whether the signal is usable, partially usable, or too ambiguous for strong inference.

When data quality appears poor, the wearable stream may still be useful, but the focus shifts. Rather than treating the output as evidence about activity, sleep, or recovery, the discussion may focus more appropriately on tracking feasibility, device burden, routine fit, or reasons for inconsistent use. In this way, signal filtering functions as an interpretive gate that protects against overreading low-quality data.

##### 3.3.2.2 Step 2: read the behavior pattern

Once the signal is judged usable, the next question becomes what kind of behavior process the pattern may plausibly reflect. This step moves from description to interpretation. COM-B offers one useful lens by asking whether the observed pattern is more consistent with limitations in capability, opportunity, or motivation (27). SCT adds a lens on confidence and mastery, particularly when repeated lapses follow missed goals (12, 20). Habit theory is useful when the pattern suggests weak cue-linked repetition, unstable routine timing, or strong dependence on favorable circumstances (21, 22).

The output of this step is not a definitive causal claim, but a working behavioral hypothesis. For example, low activity may be interpreted provisionally as a problem of routine instability rather than low motivation, or declining wear adherence may be interpreted as a signal of reduced perceived relevance rather than simple non-compliance. Contextual inquiry remains essential because the device does not reveal the life circumstances surrounding the pattern.

##### 3.3.2.3 Step 3: align a behavior change target

After a working hypothesis has been formed, the practitioner selects one primary target for the next cycle of support. The key issue is alignment since the target should match the inferred behavioral mechanism, remain feasible in context, and be specific enough to review meaningfully. This often means prioritizing a behavior process over a numeric outcome (25, 27).

Depending on the pattern, the most coherent target may involve increasing activity volume, stabilizing exercise timing, protecting sleep regularity, improving recovery rhythm, or first restoring consistency of self-monitoring itself. Where confidence is fragile or context is unstable, a smaller process target may be more coherent than an ambitious metric target.

##### 3.3.2.4 Step 4: message and micro-plan

Interpretation becomes useful only when it is translated into communication and an actionable next step. In FRAME, this involves two linked tasks starting with framing the conversation and specifying a short-horizon plan. SDT informs the communication component by emphasizing language that acknowledges perspective, preserves choice, and invites collaboration rather than imposing judgment (11, 14, 19). SCT supports planning language that builds mastery and perceived feasibility rather than performance pressure (12, 20). Table 2 provides illustrative examples of how the same wearable pattern can be communicated in either autonomy-supportive or controlling ways.

**Table 2:** Illustrative communication examples for step 4: autonomy-supportive vs. controlling framing.

Situation	Autonomy-supportive (use this)	Controlling/prescriptive (avoid this)
Low step count this week	“Your steps were lower this week. Does that fit with how the week felt? What seemed to get in the way?”	“You only reached 4,000 steps. You need to do better next week.”
Missed planned sessions	“You completed some of the plan and not all of it. What made the completed sessions more workable?”	“You had the device on, so you should have been able to follow the plan.”
Setting next week's focus	“There are a couple of reasonable directions here. Would it make more sense to work on one additional walk or on more regular bedtimes first?”	“You need to aim for 8,000 steps every day and sleep by 10 pm.”
Lapse after a strong week	“This week looks like it was harder to maintain. What changed, and what part of the previous plan still felt doable?”	“You broke the streak, so now you need to start again from the beginning.”
Irregular sleep data	“The sleep pattern looks less regular this week. Does that reflect your experience, and what do you think was driving it?”	“Your sleep efficiency is too low. You need to fix that.”
Discouragement in response to the data	“It makes sense that those numbers feel discouraging. Shall we look at what remained possible and build from there?”	“The data are clear. If results are not improving, you need to try harder.”

This table is included as an illustration of how communication style can alter the meaning and motivational impact of the same data pattern. It is not intended as a comprehensive taxonomy of effective or ineffective practitioner language.

The resulting micro-plan should identify a concrete behavior, when and where it is likely to occur, the barrier most likely to interfere, and a simple coping response if that barrier arises. In this framing, wearable feedback does not function as a verdict on past performance; it functions as a prompt for the next experiment in behavior support.

##### 3.3.2.5 Step 5: evaluate and iterate

Each support cycle ends with review and adaptation. Self-regulation provides the broad logic of comparing intention, behavior, and outcome over time, while relapse prevention helps interpret lapses as informative rather than as evidence of failure (23, 24). The review question is therefore not simply whether the number improved, but whether the selected target was feasible, whether the working hypothesis still appears plausible, and what should be maintained, simplified, scaled, or reframed next.

Process indicators may be especially informative at this stage, including consistency of completion, pattern stability, routine fit, wear adherence, and subjective response to the plan. The endpoint of one cycle therefore becomes the starting point for the next, reinforcing the iterative character of FRAME.

### 3.4 Applying FRAME: illustrative scenarios

The purpose of these scenarios is to show how the framework may organize interpretation, not to demonstrate empirical effectiveness.

#### 3.4.1 Example A: exercise routine instability in a recreational fitness context

A participant presents with wearable data showing two high-effort training sessions per week, irregular training days, declining sleep regularity, and no obvious change in an estimated fitness score. A metric-centered interpretation might focus on insufficient effort or the need for more intensity. Within FRAME, a different reading may emerge. If the signal is usable and no scoping concerns are present, the pattern may suggest routine instability and inconsistent recovery rather than lack of motivation.

Using COM-B, the main constraint may be opportunity rather than intention, particularly if work demands vary considerably across the week. Habit theory further suggests that the more relevant target may be stabilizing training timing and preserving a repeatable rhythm rather than immediately pursuing a physiological endpoint. The next target may therefore be framed as completing three planned sessions within a consistent time window, rather than “improving VO_2_max.” In this scenario, the estimated fitness score remains secondary as it may provide context, but it does not determine the behavior support response.

#### 3.4.2 Example B: activity support in a clinically adjacent lifestyle context

A patient with prediabetes presents with irregular daily step counts, prolonged sedentary periods during work hours, inconsistent sleep timing, and poor device wear on high-stress days. Within FRAME, these outputs are not interpreted as direct evidence of glycemic control. Rather, they may be read as behaviorally meaningful indicators of routine fragility, opportunity constraints, and inconsistent self-monitoring.

Using COM-B, the pattern may suggest that the immediate barrier lies more in opportunity and routine structure than in motivation. A target such as brief post-meal walking on selected working days may therefore be more coherent than a broad target of increasing overall daily steps. Sleep irregularity may be noted as potentially relevant, but not necessarily addressed within the same review cycle. In this use case, the wearable informs behavioral discussion, while clinical markers and treatment decisions remain outside the framework and within appropriate clinical judgment.

#### 3.4.3 Example C: lapse-sensitive support after an all-or-nothing cycle

A person shows a familiar pattern of several highly active days followed by multiple days of no structured activity, reduced device wear, and discouragement about having “fallen off track.” A narrow reading might equate this with poor adherence. FRAME instead encourages a more lapse-sensitive interpretation. The pattern may reflect overambitious planning, fragile confidence after perceived failure, or a lack of minimum-viable routines that can survive disruption.

SCT suggests that self-efficacy may be especially vulnerable after missed goals, while relapse-prevention principles support normalizing the lapse and learning from it rather than treating it as evidence of failure. A more coherent next step may therefore involve protecting a smaller, more repeatable version of the routine rather than attempting to restore the original aspirational plan immediately. Here, the wearable helps make the rhythm of disengagement visible, but the behavior support response depends on interpretation rather than on the metric alone.

## 4 Discussion

FRAME is proposed as a conceptual response to a practical gap in wearable-supported behavior change work in the absence of a clear interpretive bridge between consumer-generated data and behaviorally coherent support. The framework organizes a sequence of decisions that begins with data usability, moves through provisional interpretation, and culminates in short-cycle planning and iterative review. In doing so, it brings together insights from behavior change theory, engagement research, and implementation literature into a practitioner-facing workflow for real-world use (4, 5, 7, 8).

The value of FRAME lies in its translational focus, which can be clarified by comparison with existing contributions. The engagement framework of Perski and colleagues conceptualizes engagement with digital behavior change interventions as a multidimensional construct shaped by intervention, user, and contextual factors; it explains why engagement fluctuates, but it does not specify how a practitioner should act on an ambiguous or low-quality wearable signal during a consultation (1, 7). Implementation-oriented contributions, including research on integrating patient-generated data into clinical workflows, clinician-centered dashboard design, and the Wearable Activity Tracker Checklist for Healthcare (WATCH), a 12-point guide for implementing wearable activity trackers in healthcare, identifies practical considerations related to data access, workflow integration, and practitioner capability (4, 5, 9). However, these tools generally stop short of explaining how wearable data should be behaviorally interpreted once they appear in front of a practitioner. Finally, feedback-design research demonstrates that the content, framing, and timing of feedback influence outcomes independently of data access, but it has focused primarily on automated message design rather than on live consultation reasoning (25, 26). FRAME complements these strands by supplying the missing interpretive layer which is a a practitioner-facing workflow that links signal appraisal, behavioral reading, target selection, communication, and iterative review within a single model.

A further contribution of the framework is its effort to reframe the role of wearable data in practice. Rather than treating numbers as endpoints in themselves, FRAME positions them as prompts for inquiry, dialogue, and hypothesis generation. This stance may be particularly useful in settings where wearable outputs are abundant but their behavioral meaning remains uncertain (1, 2, 6). By shifting attention from metric surveillance to pattern interpretation, the framework encourages a more cautious, relational, and behavior-centered use of consumer-generated data.

The framework is intentionally scoped to contexts in which wearable outputs are used to support behavior-focused conversations. Within that scope, its primary purpose is to offer a structured heuristic for interpretation and planning rather than a direct test of intervention effectiveness. This gives FRAME a practical but bounded role by helping to clarify how wearable data may contribute to support decisions while leaving space for clinical judgment, contextual adaptation, and future empirical refinement.

In terms of applicability, FRAME is intended primarily for practitioners who conduct behavior-focused conversations supported by consumer wearable data, including dietitians and nutrition practitioners, exercise and fitness professionals, health and wellness coaches, health-promotion nurses, and clinicians providing lifestyle support in primary care or clinically adjacent settings. On the user side, the framework is most applicable to generally healthy adults and to individuals in lifestyle-support contexts, such as those with elevated cardiometabolic risk, in whom wearable data inform behavioral discussion rather than diagnosis or treatment. It is least applicable where consultation time does not permit collaborative review, where users do not generate interpretable data, or where high-acuity monitoring demands clinical-grade measurement. Whether FRAME functions equally well across these practitioner groups, and across coaching vs. clinical consultation styles, is an empirical question for feasibility work.

### 4.1 Limitations

Several limitations should be acknowledged. First, FRAME is a conceptual framework and has not yet been empirically evaluated. Its feasibility, usability, and effects on practitioner decision-making, consultation quality, or behavioral outcomes remain unknown. At this stage, the framework should be understood as a structured hypothesis about how wearable-supported behavior conversations may be organized rather than as a validated protocol.

Second, the framework was developed primarily for consumer wearables used in health-promotion, wellness, recreational fitness, and clinically adjacent lifestyle-support contexts. It is not intended for high-acuity remote monitoring, diagnostic interpretation, or contexts in which wearable data independently determine clinical decisions. Where consumer and clinical uses intersect, the framework's logic may still be informative, but only as a secondary interpretive aid alongside appropriate clinical judgment.

Third, the narrative synthesis prioritized conceptual relevance over methodological comprehensiveness. Sources were included because they clarified recurring interpretive problems or helped justify framework components, not because they constituted a formally exhaustive review set. The framework therefore reflects synthesis judgment and is vulnerable to omission of relevant evidence outside the selected corpus which is a limitation acknowledged in prior framework-oriented work in this field (7, 8).

Fourth, the framework does not yet account fully for variation in practitioner training, consultation length, organizational workflow, device ecosystem, or user population. The same wearable pattern may be interpreted differently in coaching, dietetic, community, or hybrid care settings, and further work is needed to determine whether FRAME is equally usable across these contexts.

## 5 Conclusion

Consumer wearables generate data, but they do not independently generate interpretation, meaning, or behavior change. The practical challenge for practitioners is therefore not merely how to access wearable outputs, but how to use them without overinterpreting them. FRAME addresses this challenge by offering a theory-informed conceptual workflow for moving from signal appraisal to behavioral interpretation, target selection, communication, and iterative review. Its contribution is not a new theory of behavior change, but a pragmatic, device-agnostic decision pathway for practitioners working across health-promotion, wellness, and recreational fitness contexts. Theoretically, the framework demonstrates how complementary behavior change perspectives can be organized by function around a shared practical problem, rather than treated as competing explanatory accounts. Practically, it offers practitioners a usable structure for wearable-informed consultations, and offers wearable users the prospect of conversations in which their data serve as a starting point for collaborative problem-solving rather than as a verdict on adherence.

Feasibility and usability testing in practitioner settings is the necessary next step, alongside pilot work examining whether FRAME improves the behavioral coherence of wearable-supported consultations across diverse populations and care contexts. As the wearable landscape continues to evolve, the framework's interpretation logic will require ongoing revision to remain relevant to the data environments practitioners actually encounter.

## Data Availability

The original contributions presented in the study are included in the article/Supplementary Material, further inquiries can be directed to the corresponding author.
